# Ca^2+^/calmodulin-dependent protein kinase IIγ enhances stem-like traits and tumorigenicity of lung cancer cells

**DOI:** 10.18632/oncotarget.3866

**Published:** 2015-04-19

**Authors:** Shoujie Chai, Xia Xu, Yongfang Wang, You Zhou, Chenchen Zhang, Yiming Yang, Ying Yang, Haiyan Xu, Rongzhen Xu, Kai Wang

**Affiliations:** ^1^ Department of Respiratory Medicine, Second Affiliated Hospital, Zhejiang University School of Medicine, Hangzhou, China; ^2^ Department of Hematology, Second Affiliated Hospital, Zhejiang University School of Medicine, Hangzhou, China

**Keywords:** CaMKIIγ, stem-like, tumorigenicity, lung cancer

## Abstract

Highly tumorigenic stem-like cells, considered tumor-initiating cells (TICs), are the main cause of lung cancer initiation, relapse, and drug resistance. In this study, we identified that Ca^2+^/calmodulin-dependent protein kinase IIγ (CaMKIIγ) was aberrantly expressed in highly tumorigenic stem-like lung cancer cells, and was also correlated with poor prognosis in human lung cancer. Functionally, CaMKIIγ enhanced stem-like traits and the tumorigenicity of lung cancer cells in an Akt- and β-catenin-dependent manner. In addition, we found that CaMKIIγ upregulated Oct4 expression via Akt-mediated histone acetylation. Taken together, our findings reveal a critical role of CaMKIIγ in regulating the stemness and tumorigenicity of lung cancer cells and offer a promising therapeutic target for TICs.

## INTRODUCTION

Phenotypic heterogeneity of cancer cells as a consequence of genetic change and environmental differences leads to formation of a tumor ecosystem composed of various cell populations that have different functions, like tumorigenesis, metastasis, relapse, and drug resistance [1]. In the tumor ecosystem, highly tumorigenic stem-like cells, considered tumor-initiating cells (TICs) or cancer stem cells (CSCs), perform a vital role in tumor initiation and development [2]. Such a population is also responsible for tumor relapse after treatment. One notable example is lung cancer, the leading annual cause of cancer-related mortality worldwide [3]. Due to the success of therapy targeted to driver genes, many patients with lung cancer have a good initial response to therapy; however, most experience a relapse within one year [4, 5]. Thus, specifically targeting the highly tumorigenic stem-like cell population has recently been suggested as a new approach to treat lung cancer.

Transcription factors that are involved in cellular reprogramming leading to generation of embryonic stem-cell-like induced pluripotent stem cells (iPSCs) [6] have been shown to be associated with TIC properties. For example, ectopic expression of Oct4, Nanog, or c-Myc in lung cancer cells significantly increased the CD133-expressing subpopulation, oncosphere formation, tumorigenicity, and drug resistance, while knockdown produced the opposite effect, supporting the notion that iPSC factors play an essential role in high tumorigenicity and stem-like traits [7-9]. In diverse human epithelial cancers, iPSC factors are aberrantly activated or expressed, and accompany high malignancy and poor prognosis [10, 11]. However, the regulatory mechanism of abnormal activation and expression of iPSC factors in cancers is still unclear.

Ca^2+^/calmodulin-dependent kinase II (CaMKII) is a multifunctional serine/threonine kinase, consisting of four homologous (CaMKIIα/β/γ/δ). Our previous papers identified CaMKIIγ as a critical regulator of cell growth and survival in leukemia, lung cancer, and liver cancer [12-14]. In our recent study, CaMKIIγ was preferentially expressed in TICs and associated with the β-catenin, Stat3, and NF-κB signal pathways [12, 14]. However, the precise function and molecular mechanism of CaMKIIγ in stemness and tumorigenesis is unknown.

These exciting findings prompted us to investigate whether CaMKIIγ is necessary for stem-like and tumorigenic traits in cancers. The current study demonstrated that CaMKIIγ was essential for maintaining stem-like properties and the tumorigenicity of lung cancer cells. We found that CaMKIIγ was highly activated and expressed in highly tumorigenic and stem-like cells enriched from culture conditioned for lung cancer oncospheres. *In vitro* and *in vivo* assays indicated that CaMKIIγ was required for stem-like and tumorigenic characteristics of lung cancer cells. Next, we observed that CaMKIIγ enhanced stem-like traits, including the expression of iPSC factors and formation of oncospheres, in an Akt- and β-catenin-dependent manner. Surprisingly, our results revealed that CaMKIIγ regulated Akt-mediated histone acetylation of iPSC factor Oct4 to improve its expression. These observations highlight the importance of CaMKIIγ in regulating the stemness and tumorigenesis of lung cancer cells, illustrate a novel epigenetic regulation of Oct4, and offer a new approach to target TICs in lung cancer.

## RESULTS

### Lung cancer oncospheres display stem-like and highly tumorigenic characteristics

**Figure 1:**
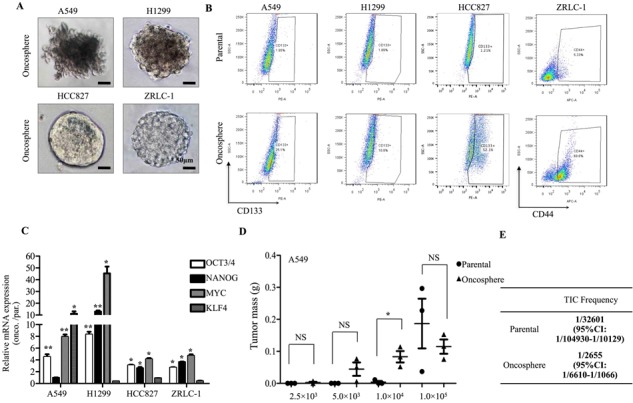
Lung cancer oncospheres exhibit stem-like and highly tumorigenic features **A.** Phase-contract micrograph of oncospheres derived from A549, H1299, HCC827, and ZRLC-1 lung cancer cell lines. Scale bar, 50 μm. **B.** Representative FCM plots for CD133 or CD44 expression and quantification of the CD133+ population in A549, H1299, and HCC827 parental or oncosphere cells, and CD44+ population in ZRLC-1 parental or oncosphere cells. **C.** Relative gene expression of OCT4, NANOG, MYC, and KLF4 in indicated oncosphere cells by real-time PCR. Data are expressed as fold of parental cells ± SEM of *p* = 3 independent cell dishes per condition. *p < 0.05, **p < 0.01 versus parental cells. **D.** A549 parental or oncosphere cells were separately injected subcutaneously into NOD/SCID mice. Data are expressed as mean ± SEM of *n* = 3 mice per group. *p < 0.05. NS: no significance. Tumor incidence is displayed on the graph. **E.** TIC frequency of A549 parental or oncosphere cells is measured by LDA *in vivo*.

Previous studies have shown that highly tumorigenic stem-like cells, also called TICs, can be enriched in serum-free medium with low adherence [15]. This conditional culture induces highly tumorigenic cells to form oncospheres within one or two weeks, while it inhibits the growth of less tumorigenic cells. We harvested oncospheres from three lung cancer cell lines (A549, H1299, and HCC827) and one primary lung cancer sample (ZRLC-1; Figure 1A). To evaluate the stem-like potential of lung cancer oncospheres, we firstly detected the fraction of stem-like surface-marker-positive populations, like CD133^+^ or CD44^+^ cells, by flow cytometry. Stem-like markers varied in different lung cancer cell lines and samples [2, 16, 17]. Our previous study indicated that CD133 was the potential stem-like marker in A549, H1299, and HCC827 cells, while CD44 was the marker in ZRLC-1 cells. We observed that lung cancer oncospheres contained higher percentages of CD133^+^ or CD44^+^ cells than did parental cells (Figure 1B & S1A). Second, we used real-time PCR to determine the mRNA expression for induced pluripotent stem cell (iPSC) factors, including OCT4, MYC, KLF4, and NANOG, which were associated with a stem-like phenotype. Results revealed that lung cancer oncospheres showed enhanced expression of these iPSC factors (Figure 1C). To identify the highly tumorigenic potential of lung cancer oncospheres, we subcutaneously implanted A549 oncosphere cells or parental cells into NOD-SCID mice. As few as 2500 oncosphere cells were sufficient for tumor initiation in one out of three hosts, whereas as many as 10000 parental cells initiated one tumor in three hosts (Figure 1D & S1B). The frequency of tumor-initiating cells (TICs) was calculated by limiting dilution assay at approximately 1/2655 for oncosphere cells and 1/32601 for parental cells (Figure 1E). Similar results were obtained in ZRLC-1 cells (Figure S1C & D).

### CaMKIIγ maintains stem-like and tumorigenic traits of lung cancer cells

**Figure 2:**
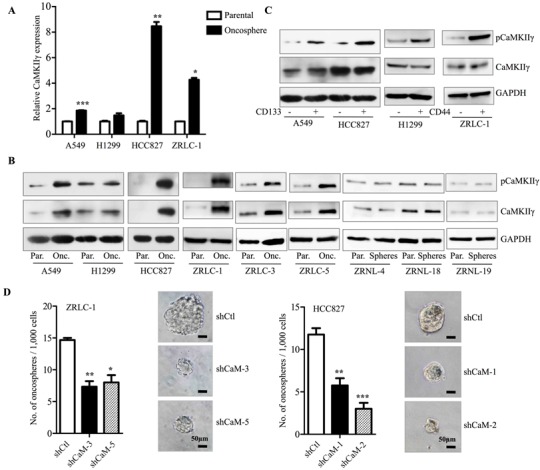

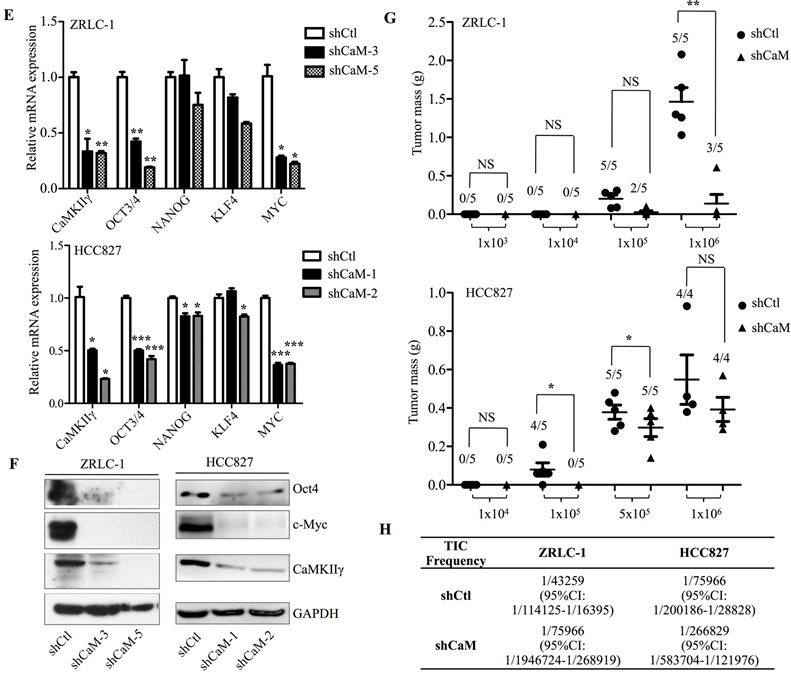
CaMKIIγ is required for maintenance of stem-like and tumorigenic properties **A.** Relative gene expression of CaMKIIγ in indicated parental or oncosphere cells by real-time PCR. Data are expressed as mean ± SEM of *n* = 3 independent cell dished per condition. *p < 0.05, **p < 0.01, ***p < 0.001 versus parental cells. **B.** Detection of activated (Phosphorylation of Ser287) and total CaMKIIγ protein level by western blots in parental or oncosphere cells from lung cancer cells (A549, H1299 and HCC827), primary lung cancer cells (ZRLC-1, ZRLC-3 and ZRLC-5) or primary normal lung cells (ZRNL-4, ZRNL-18 and ZRNL-19). **C.** Detection of activated and total CaMKIIγ protein level by western blots in sorted lung cancer cells. Quantitative analysis of oncosphere formation by 1000 control (shCtl) and CaMKIIγ knockdown (shCaM) ZRLC-1 or HCC827 cells. Data are expressed as mean ± SEM of *n* = 3 independent cell dishes per condition. *p < 0.05, **p < 0.01, ***p < 0.001 versus shCtl cells. **D.** Relative gene expression of OCT4, NANOG, MYC, and KLF4 in shCtl and shCaM ZRLC-1 or HCC827 cells by real-time PCR. Data are expressed as mean ± SEM of *n* = 3 independent cell dishes per condition. *p < 0.05, **p < 0.01, ***p < 0.001 versus shCtl cells. **E.** Detection of Oct4, c-Myc, CaMKIIγ, and GAPDH protein by western blots in shCtl and shCaM ZRLC-1 or HCC827 cells. **F.** ShCtl and shCaM ZRLC-1 or HCC827 cells were separately injected subcutaneously into nude mice. Data are expressed as mean ± SEM of *n* = 5 mice per group. *p < 0.05, **p < 0.01. NS: no significance. Tumor incidence is displayed on the graph. **G.** TIC frequency of shCtl and shCaM ZRLC-1 or HCC827 cells is measured by LDA *in vivo*.

**Figure 3:**
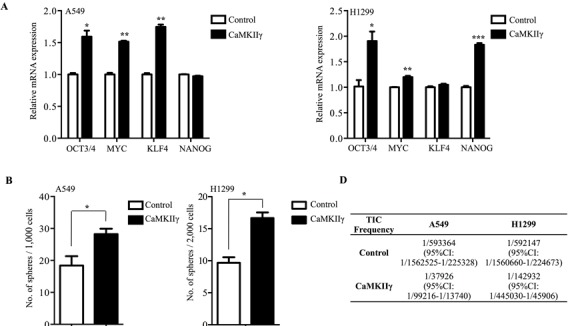

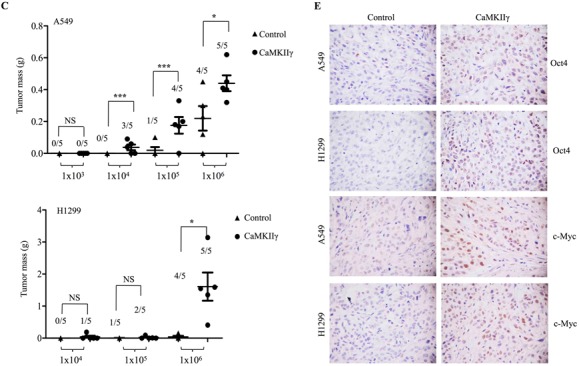
Ectopic expression of CaMKIIγ promotes stem-like and tumorigenic traits **A.** Relative gene expression of OCT4, NANOG, MYC, and KLF4 in A549 or H1299 cells stably expressing control or CaMKIIγ by real-time PCR. Data are expressed as mean ± SEM of *n* = 3 independent cell dishes per condition. *p < 0.05, **p < 0.01, ***p < 0.001 versus control cells. **B.** Quantitative analysis of oncosphere formation by 1000 or 2000 indicated cells. Data are expressed as mean ± SEM of *n* = 3 independent cell dishes per condition. *p < 0.05 versus control cells. **C.** A549 or H1299 cells stably expressing control or CaMKIIγ were separately injected subcutaneously into nude mice. Data are expressed as mean ± SEM of *p* = 5 mice per group. *p < 0.05, **p < 0.01. NS: no significance. Tumor incidence is displayed on the graph. **D.** TIC frequency of A549 or H1299 cells stably expressing control or CaMKIIγ is measured by LDA *in vivo*. **E.** Histological analysis of xenograft tumors from Figure 3C.

CaMKIIγ is required for the growth and survival of non-small cell lung cancer cells and promotes colony formation [14]. To assess the potential role of CaMKIIγ in stem-like traits and tumorigenicity, we first tested mRNA and protein expression in lung cancer oncospheres. A549 and H1299 oncospheres displayed a moderate increase in activated and total CaMKIIγ expression compared with parental cells, while HCC827, ZRLC-1, ZRLC-3 and ZRLC-5 cells exhibited significantly enhanced expression (Figure 2A & 2B). To evaluate whether CaMKIIγ was increased in normal stem-like cells, we enriched sphere cells from primary normal lung cells, ZRNL-4, ZRNL-18 and ZRNL-19, in serum-free medium with low adherence and observed that CaMKIIγ was not highly expressed or activated in normal lung sphere cells (Figure 2B). In addition, CD133^+/hi^ or CD44^+/hi^ cells sorted from four different lung cancer cells also showed higher activation of CaMKIIγ than did CD133^−/low^ or CD44^−/low^ cells (Figure 2C & S2A). Then, we depleted CaMKIIγ in ZRLC-1 and HCC827 cells for an *in vitro* assay of stem-like potential and an *in vivo* assay of tumorigenicity. We found that CaMKIIγ was necessary for oncosphere formation in serum-free and low adherent culture (Figure 2D). Additionally, CaMKIIγ knockdown sharply decreased expression of two iPSC factors, Oct4 and c-Myc, at both mRNA and protein levels (Figure 2E & 2F). Importantly, tumorigenicity was also significantly inhibited upon stable knockdown of CaMKIIγ in ZRLC-1 and HCC827 cells (Figure 2G & S2B). Based on the limiting dilution assay, TICs frequency of CaMKIIγ-deleted cells was 1/75966 for ZRLC-1 (1/43259 for control cells) and 1/266829 for HCC827 (1/75966 for control cells; Figure 2H). Our results suggested that CaMKIIγ is required for stem-like traits and tumorigenicity of lung cancer cells. To test whether ectopic expression of CaMKIIγ promotes these properties, we overexpressed CaMKIIγ in A549 and H1299 cells. CaMKIIγ overexpression significantly increased oncosphere formation in conditioned culture (Figure 3B). Similarly, expression of two iPSC factors, Oct4 and c-Myc, was enhanced and accompanied by CaMKIIγ overexpression (Figure 3A). In the *in vivo* assay, we observed that CaMKIIγ promoted tumorigenicity in A549 and H1299 cells (Figure 3C & S3A), and upregulated TICs frequency (Figure 3D). Sequential histological analysis also demonstrated that positively stained cells of Oct4 and c-Myc were significantly accumulated in CaMKIIγ-overexpressed xenograft tumors, compared with control tumors (Figure 3E).

### CaMKIIγ inhibitor impairs stem-like properties and tumorigenicity of lung cancer cells

**Figure 4:**
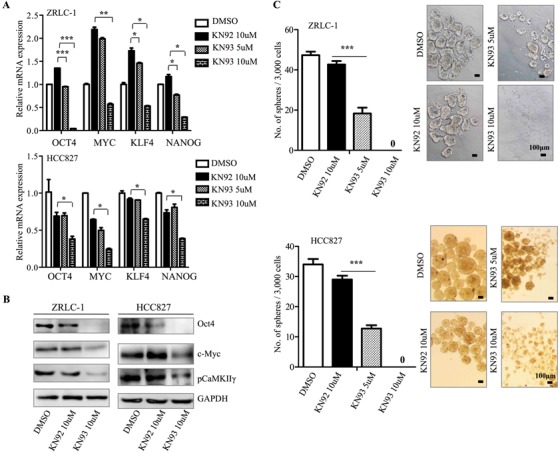

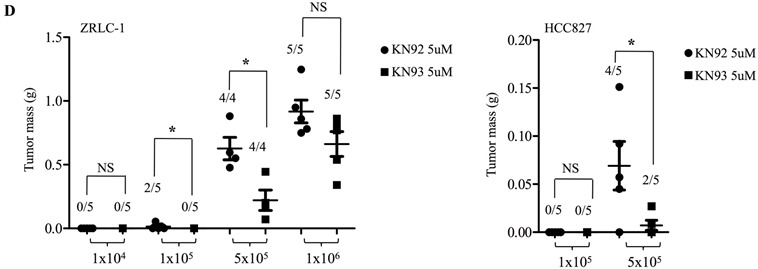
CaMKIIγ inhibitor iIrreversibly impairs lung TICs **A.** Relative gene expression of OCT4, NANOG, MYC, and KLF4 in ZRLC-1 or HCC827 cells treated with DMSO, KN92, or KN93. Data are expressed as mean ± SEM of *n* = 3 independent cell dishes per condition. *p < 0.05, **p < 0.01, ***p < 0.001 versus cells treated with KN92. **B.** Detection of Oct4, c-Myc, pCaMKIIγ, and GAPDH protein by western blots in ZRLC-1 or HCC827 cells treated with DMSO, KN92, or KN93. **C.** Quantitative analysis of oncosphere formation by 3000 ZRLC-1 or HCC827 cells treated with DMSO, KN92, or KN93. Data are expressed as mean ± SEM of *n* = 3 independent cell dishes per condition. *p < 0.05 versus cells treated with KN92. **D.** ZRLC-1 or HCC827 cells pre-treated with KN92 or KN93 were separately injected subcutaneously into nude mice. Data are expressed as mean ± SEM of *n* = 5 mice per group. *p < 0.05, **p < 0.01. NS: no significance. Tumor incidence is displayed on the graph.

To investigate whether KN93, an effective inhibitor of CaMKII kinase activity, impairs stem-like and tumorigenic properties, we treated lung cancer cells with KN93 or KN92 (a structural analog with no corresponding inhibitory effect) for *in vitro* and *in vivo* assays. In ZRLC-1 and HCC827 cells, treatment with KN93 at 10 μM (IC_50_) yielded a significant decrease in the mRNA and protein expression of iPSC factors, especially Oct4 and c-Myc (Figure 4A & 4B). Surprisingly, 10 μM KN93 completely inhibited oncosphere formation in both cell lines. A subsequent assay showed that 5 μM (half IC_50_) KN93 still had an inhibitory effect on oncosphere formation (Figure 4C). To determine whether KN93-mediated impairment of tumorigenic potential was transient or irreversible, we exposed ZRLC-1 and HCC827 cells to KN93 for four days, removed the inhibitor, and subcutaneously injected the cells into nude mice. Tumorigenicity was significantly reduced following KN93 treatment, compared with KN92 treatment (Figure 4D & S4A). Therefore, our results support that KN93 impairs stem-like properties, including the expression of iPSC factors and oncosphere formation, and the transient inhibition of CaMKIIγ also irreversibly reduces tumorigenic potential.

### CaMKIIγ is correlated with the expression of iPSC factors and prognosis in human lung cancer

**Figure 5:**
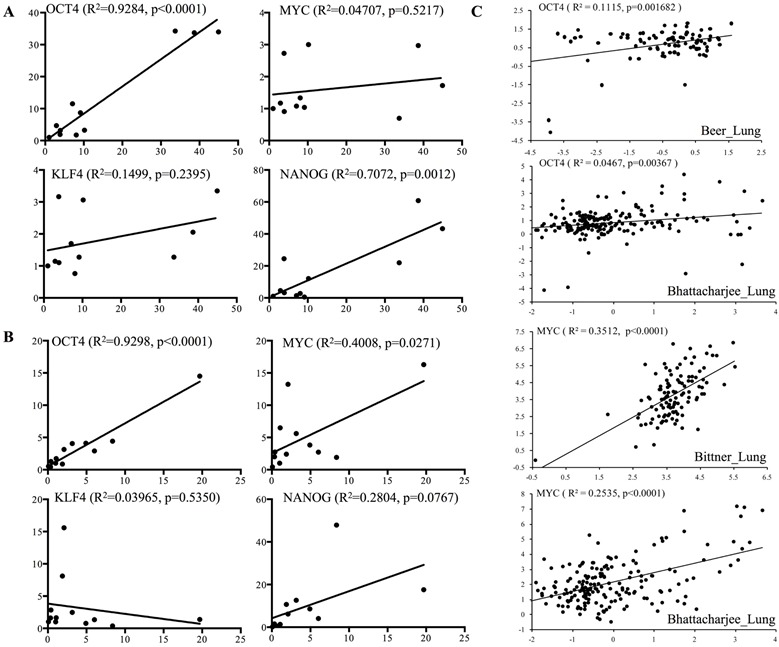

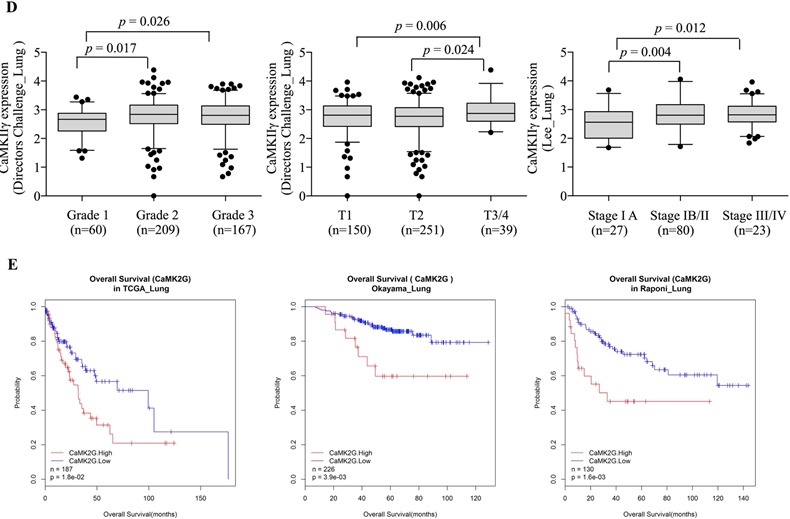
CaMKIIγ expression is correlated with OCT4 and prognosis in human lung cancer **A.** Correlation between CaMKIIγ and iPSC factors (OCT4, MYC, KLF4, or NANOG) is detected by real-time PCR for gene expression in 11 lung cancer cell lines. X axis stands for CaMKIIγ expression and Y axis stands for iPSC factors expression. **B.** Correlation between CaMKIIγ and iPSC factors (OCT4, MYC, KLF4, or NANOG) is detected by real-time PCR for gene expression in 12 lung cancer samples. X axis stands for CaMKIIγ expression and Y axis stands for iPSC factors expression. **C.** Correlation between CaMKIIγ and iPSC factors (OCT4, MYC) is analyzed from Oncomine^®^ microarray data. X axis stands for CaMKIIγ expression and Y axis stands for OCT4 or MYC expression. **D.** Corrlation between CaMKIIγ expression and clinicopathological characteristics including pathological grade, tumor stage and clinical stage is analyzed from Oncomine^®^ microarray data. **E.** Survival analysis of lung cancer patients with high or low CaMKIIγ expression, according to Oncomine® microarray data.

To assess the relevance of CaMKIIγ and iPSC factors in human lung cancer, we examined the mRNA expression of CaMKIIγ and iPSC factors in eleven lung cancer cell lines and twelve lung cancer samples. In cancer cell lines, CaMKIIγ was positively correlated with OCT4 and NANOG expression, but was not correlated with MYC or KLF4 (Figure 5A). In cancer samples, CaMKIIγ was positively correlated with OCT4 and MYC (Figure 5B). To further examine the relationship between CaMKIIγ and two iPSC factors, we downloaded the expression data of CaMKIIγ, OCT4, and MYC in lung cancer from the Oncomine^®^ microarray database for Pearson correlation analysis. Our results revealed that CaMKIIγ was positively correlated with MYC expression in most studies, and CaMKIIγ was positively correlated with OCT4 in some studies (Figure 5C & S5A). Furthermore, we obtained the clinical data and CaMKIIγ expression of lung cancer from Oncomine^®^. Results indicated that patients with high pathological grade or high clinical stage showed higher expression of CaMKIIγ than ones with low grade or low stage (Figure 5D & S5B). Besides, CaMKIIγ expression had no correlation with cancer type, like adenocarcinoma, squamous cell carcinoma or large cell carcinoma (Figure S5C). Surprisingly, in three groups of Oncomine^®^ data, we observed that patients with high CaMKIIγ expression displayed a significant poor prognosis (Figure 5E).

### CaMKIIγ enhances stem-like traits in an Akt- and β-catenin-dependent manner

**Figure 6:**
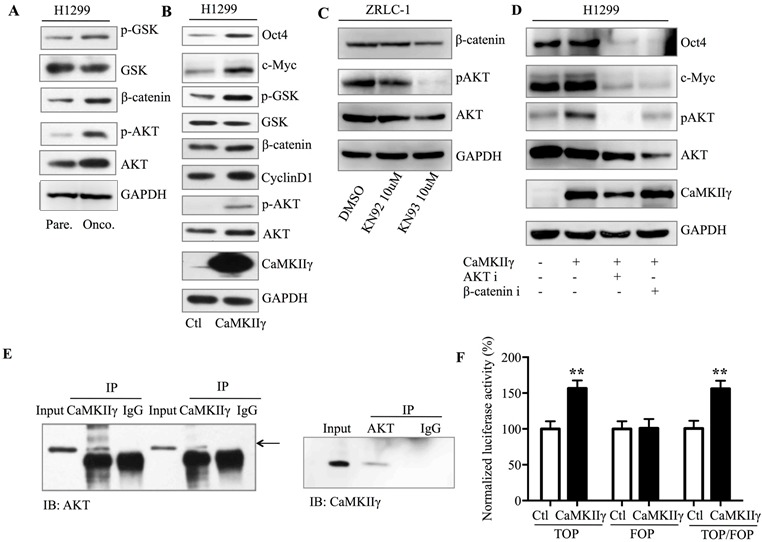

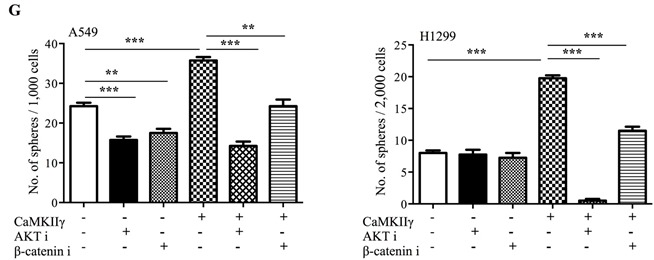
CaMKIIγ enhances stem-like traits in an Akt- and β-catenin-dependent manner **A.** Detection of Akt and β-catenin signals in H1299 parental or oncosphere cells by western blots. **B.** Detection of Akt and β-catenin signals in H1299 cells tranduced with control or CaMKIIγ vector by western blots. **C.** Detection of Akt and β-catenin signals in ZRLC-1 cells treated with DMSO, KN92, or KN93 by western blots. **D.** Detection of Oct4 and c-Myc in CaMKIIγ-overexpressed H1299 cells treated with Akt or β-catenin inhibitor by western blots. **E.** Lysates of CaMKIIγ-overexpressed A549 and H1299 cells were incubated with CaMKIIγ or Akt antibody for immunoprecipitation and the immune complex was then purified, separated by SDS-PAGE, and immunoblotted with Akt or CaMKIIγ antibody. **F.** Dual luciferase reporter assay of Wnt pathway (TOP, FOP, and the ratio of TOP/FOP) in control or CaMKIIγ-overexpressed H1299 cells. Data are expressed as mean ± SEM of *n* = 4 independent cell dishes per condition. **p < 0.01. **G.** Quantitative western blot analysis of oncosphere formation by control or CaMKIIγ-overexpressed A549 or H1299 cells treated with Akt or β-catenin inhibitor. Data are expressed as mean ± SEM of *n* = 4 independent cell dishes per condition. *p < 0.05, **p < 0.01, ***p < 0.001.

The Akt-Oct4 circuit and Wnt-β-catenin-c-Myc pathway have been implicated as important regulators of stemness [18, 19]. The above results prompted us to investigate the role of Akt and β-catenin signals in the enhancement of stem-like traits, mediated by CaMKIIγ. We observed that Akt and β-catenin signals were activated in H1299 oncospheres (Figure 6A). Furthermore, overexpression of CaMKIIγ activated Akt and β-catenin signals in H1299 cells (Figure 6B), while CaMKIIγ inhibitor reduced the activation in ZRLC-1 cells (Figure 6C). To determine whether CaMKIIγ-activated Akt and β-catenin signals were involved in the regulation of Oct4 and c-Myc expression, we treated CaMKIIγ-overexpressed cells with inhibitors of Akt and β-catenin signals. We found that CaMKIIγ-overexpressed cells displayed a significant decrease in Oct4 and c-Myc expression following treatment with Akt and β-catenin inhibitors (Figure 6D). Our results indicated that both Akt and β-catenin signals are involved in the regulation of Oct4 and c-Myc expression, and there may be crosstalk between the Akt and β-catenin pathways. We also used co-immunoprecipitation (co-IP) to examine whether CaMKIIγ interacted with Akt, and to test our Scansite prediction that CaMKIIγ could phosphorylate Akt at the Ser473 site. We discovered that CaMKIIγ interacted with Akt in lung cancer cells (Figure 6E). In our previous study, we also showed that CaMKIIγ interacted with β-catenin [12]. These data suggested that Akt and β-catenin might be substrates of CaMKIIγ kinase. To test whether CaMKIIγ-regulated Akt and β-catenin signals participated in oncosphere formation, we exposed CaMKIIγ-overexpressed cells to Akt and β-catenin inhibitors for an *in vitro* assay. The data showed that Akt and β-catenin inhibitors antagonized the enhancement of oncosphere formation mediated by CaMKIIγ in both A549 and H1299 cells (Figure 6G). To investigate the relationship between CaMKIIγ and other pathways involved in stemness, like Notch, or Hedgehog pathway, we used dual luciferase reporter assay to detect the effect of CaMKIIγ on these pathways and found that CaMKIIγ overexpression promoted the expression of TOP and RBP-JK reporter which represented Wnt and Notch pathway, while it had no influence on GLI reporter for Hedgehog pathway (Figure 6E & S6B).

### CaMKIIγ regulates Akt-mediated histone acetylation of OCT4

**Figure 7:**
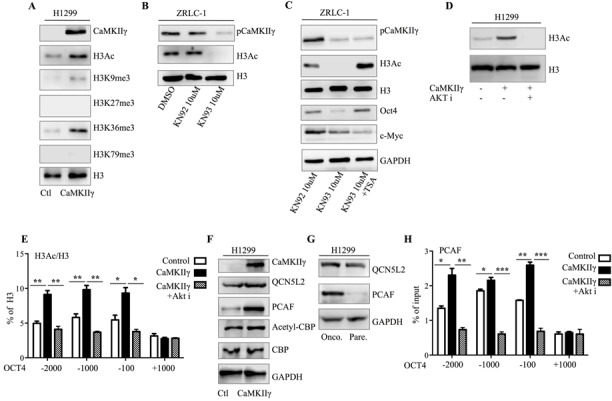
CaMKIIγ regulates Akt-mediated histone acetylation of OCT4 **A.** Detection of acetylation and methylation of histone in control or CaMKIIγ-overexpressed H1299 cells by western blots. **B.** Detection of acetylated histone3 (H3Ac) in ZRLC-1 cells treated with DMSO, KN92, or KN93 by western blots. **C.** Detection of H3Ac, Oct4, and c-Myc in KN93-treated ZRLC-1 cells cultured with TSA by western blots. **D.** Detection of H3Ac in control or CaMKIIγ-overexpressed H1299 cells treated with Akt inhibitor by western blots. **E.** CHIP-enriched DNA from control or CaMKIIγ-overexpressed H1299 cells treated with Akt inhibitor using anti-H3Ac antibody is amplified by real-time PCR, depending on primers designed according to the transcription regulatory region of OCT4. Data are expressed as enrichment assessed relative to the input DNA ± SEM. *p < 0.05, **p < 0.01, ***p < 0.001. **F.** Detection of histone acetylation transferases in control or CaMKIIγ-overexpressed H1299 cells by western blots. **G.** Detection of histone acetylation transferases in H1299 parental or oncosphere cells by western blots. (H) CHIP-enriched DNA from control or CaMKIIγ-overexpressed H1299 cells treated with Akt inhibitor using anti-PCAF antibody is amplified by real-time PCR, depending on primers designed according to the transcription regulatory region of OCT4. Data are expressed as enrichment assessed relative to the input DNA ± SEM. *p < 0.05, **p < 0.01, ***p < 0.001.

**Figure 8:**
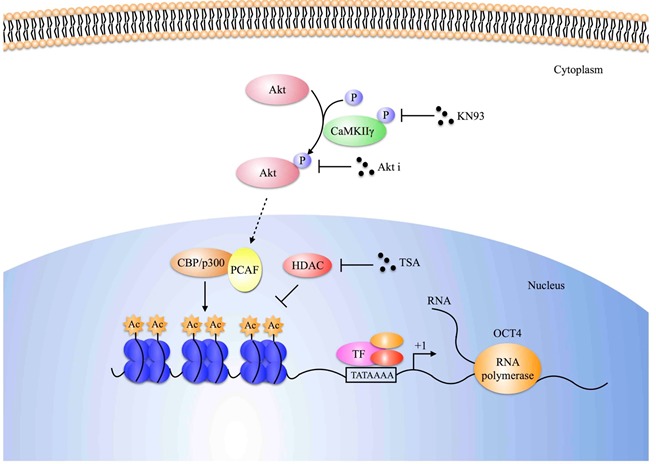
Schematic model of epigenetic regulation of Oct4 expression Schematic representation of how CaMKIIγ regulates Oct4 expression in lung cancer cells. CaMKIIγ enhances Oct4 expression depending on the increase of histone acetylation, which is mediated by acetylation transferases, especially for PCAF. Besides, Akt, activated by CaMKIIγ, is involved in this epigenetic regulation, which can be suppressed by Akt inhibitor.

A recent paper demonstrated that Akt activation promotes histone acetylation in cancer cells [20]. We speculated that CaMKIIγ-induced Akt activation could enhance histone acetylation to improve transcription of OCT4 and MYC. First, we tested common modifications of histone in CaMKIIγ-overexpressed or -inhibited cells, and observed that histone was dominantly acetylated or de-acetylated when CaMKIIγ was overexpressed or inhibited (Figure 7A & 7B). Second, to identify whether de-acetylation mediated by KN93 could be secured by histone deacetylase inhibitor TSA, we treated ZRLC-1 cells with KN93 or KN93+TSA, and then tested Oct4 and c-Myc expression using western blots. Surprisingly, Oct4 expression reduced by KN93 was rescued following TSA treatment, while TSA had no effect on c-Myc expression (Figure 7C). Third, to further examine whether Akt was involved in CaMKIIγ-triggered histone acetylation, we exposed CaMKIIγ-overexpressed cells to Akt inhibitor and found that CaMKIIγ-elevated histone acetylation was antagonized by Akt in H1299 cells (Figure 7D). Fourth, to demonstrate that CaMKIIγ-regulated Oct4 expression was associated with histone acetylation, we used chromatin immunoprecipitation (CHIP) technology to quantify histone acetylation at the OCT4 gene in control cells, CaMKIIγ-overexpressed cells, or CaMKIIγ-overexpressed cells treated with Akt. Histone was significantly acetylated in CaMKIIγ-overexpressed cells; acetylation decreased following Akt inhibitor treatment (Figure 7E). Next, we detected common histone acetyltransferases QCN5L2, PCAF, and CBP, and observed that PCAF was dominantly expressed in CaMKIIγ-overexpressed cells and oncosphere cells (Figure 7F & 7G). Finally, we used CHIP-PCR to quantify the enrichment of PCAF at the OCT4 gene. CaMKIIγ overexpression promoted PCAF binding to OCT4, which was reduced by Akt inhibitor (Figure 7H). Collectively, we concluded that CaMKIIγ regulates histone acetylation to enhance Oct4 expression through Akt, as shown in Figure 8.

## DISCUSSION

The current study defines a critical role of CaMKIIγ in the stemness and tumorigenesis of lung cancer cells. Although CSCs and TICs have not previously been identified in lung cancer, several studies of other cancers have identified a small subpopulation of highly tumorigenic and stem-like cells that are considered CSCs or TICs. Such a subpopulation was significantly associated with poor prognosis, drug resistance, and tumor relapse, and is becoming a highlighted target for the treatment of low-differentiated, drug-resistant, or recurrent lung cancer.

Glycine decarboxylase (GLDC), PKCι, Notch3, and integrin β_3_-KRAS-RalB have been shown to play important roles in the acquisition and maintenance of stemness in lung cancer [2, 15, 16, 21]. Still, most targets cannot be translated into clinical application, due to similar expression or activation in normal stem cells. Here, we identified CaMKIIγ as a potential target for stemness and tumorigenesis.

Our previous study demonstrated that CaMKIIγ was aberrantly activated and expressed in lung cancer tissue, and that normal hematopoietic stem cells expressed CaMKIIγ minimally or not at all [12, 14]. Furthermore, we found that CaMKIIγ was preferentially expressed in highly tumorigenic and stem-like cells, which were regarded as CSCs or TICs, compared with parental cells. These findings drove us to make a detailed study of the function and regulatory mechanism of CaMKIIγ in stemness and tumorigenesis.

Highly tumorigenic and stem-like cells are commonly sorted or enriched by stem cell markers or non-marker technology, like oncosphere formation or drug resistance. Due to tumor heterogeneity, a standard set of stem cell markers has not been identified for lung cancer. CD166^+^, CD133^+^, CD44^+^, and CD24^+^ITGB4^+^NOTCH^hi^ cells have been labeled as CSCs or TICs in different studies [2, 16], but few of these studies could be repeated in lung cancer cell lines or samples. In addition, highly tumorigenic and stem-like cells can be enriched following serum-free and low adherent culture. After one or two weeks of conditional culture, lung cancer oncospheres formed and were collected for *in vitro* and *in vivo* assays. In our study, we indicated that oncosphere cells displayed stem-like traits, including higher expression of stem cell markers and iPSC factors, and higher tumorigenicity compared with parental cells. Importantly, our results revealed that CaMKIIγ was highly activated and expressed in lung cancer oncospheres, which were enriched from lung cancer cell lines and primary lung cancer cells.

Next, we found that knockdown of CaMKIIγ in lung cancer cells significantly reduced the expression of iPSC factors (Oct4 and c-Myc), oncosphere formation, and tumorigenicity. This suggested that CaMKIIγ was necessary for maintaining stem-like and tumorigenic properties. In contrast, ectopic expression of CaMKIIγ enhanced these traits. Further, we treated lung cancer cells with CaMKII inhibitor KN93, and observed that expression of iPSC factors and ability of oncosphere formation sharply decreased following KN93 treatment. Moreover, inhibition of CaMKIIγ activity irreversibly impaired tumorigenic potential. Thus, we concluded that CaMKIIγ maintains stem-like and tumorigenic characteristics, depending on kinase activity and that iPSC factors, especially Oct4 and c-Myc, may be the important effectors regulated by CaMKIIγ.

The Akt-Oct4 circuit and Wnt-β-catenin-c-Myc pathway have been implicated as important regulators of stemness. In the present study, we discovered that Akt and β-catenin signals were indeed involved in the enhancement of Oct4 and c-Myc expression and oncosphere formation mediated by CaMKIIγ. Additionally, Akt and β-catenin signals may have a crosstalk on regulation of Oct4 and c-Myc expression. In our previous study, we demonstrated that CaMKIIγ interacts with β-catenin and promotes its nuclear location. Here, we also certified that CaMKIIγ interacted with Akt and used Scansite, an online tool, to predict that CaMKIIγ could phosphorylate Akt at the Ser 473 site (http://scansite.mit.edu). Therefore, we draw a conclusion that CaMKIIγ enhances stem-like traits, consisting of Oct4 and c-Myc expression and oncosphere formation, in an Akt- and β-catenin-dependent manner.

Emerging evidence indicates that Akt phosphorylates Oct4 at threonine 235 and facilitates its nuclear localization. In the present study, we investigated how Akt functioned with Oct4, except for direct activation, and whether Akt could regulate Oct4 expression. A recent paper indicated that Akt stimulated histone acetylation by increasing acetyl coenzyme A (acetyl-CoA) availability [20]. We speculated that CaMKIIγ-induced Akt activation could enhance histone acetylation, to improve the transcription of OCT4. We found that CaMKIIγ overexpression increased the histone acetylation level at the OCT4 gene, while treatment with Akt inhibitor decreased this OCT4 modification. KN93, a CaMKII inhibitor, diminished acetylated histone, which was restored by the histone deacetylase inhibitor, TSA. Collectively, our data suggested that CaMKIIγ orchestrated the epigenetic regulation of Oct4 expression, depending on Akt signal.

Wnt, Notch and Hedgehog pathways have been reported as important pathways in the stemness of lung cancer [22, 23]. CaMKII is described as a mediator of non-canonical Wnt pathway [24]. Here, we investigated the crosstalk between CaMKIIγ signaling and canonical Wnt pathway. Results indicated that CaMKIIγ-Akt signaling regulates canonical Wnt pathway, while the precise mechanism needs further studies. Besides, depending on the dual luciferase reporter assay, we concluded that CaMKIIγ signaling activated Notch pathway, while it had no influence on Hedgehog pathway. The mechanism how CaMKIIγ regulates Notch pathway, needs further study. Meanwhile, we also demonstrated that CaMKIIγ-Akt epigenetically enhanced Oct4 expression via histone acetylation.

Finally, we determined whether there was a correlation between CaMKIIγ, Oct4, and human lung cancer prognosis. CaMKIIγ was positively correlated with Oct4 and c-Myc in lung cancer samples, and patients with higher expression of CaMKIIγ had significantly worse prognosis. In general, the clinical observations were in accordance with the above results. Based on our findings that CaMKIIγ maintains stem-like and tumorigenic properties, depending on Akt and β-catenin, that CaMKIIγ regulates Akt-mediated histone acetylation of Oct4, and that CaMKIIγ is positively correlated with Oct4 and poor prognosis, we propose that CaMKIIγ is a potential biomarker for highly tumorigenic and stem-like cells.

## MATERIALS AND METHODS

### Reagents and antibodies

Phospho-CaMKII (Thr287), CaMKIIγ, and GAPDH antibodies were obtained from Santa Cruz Biotechnology. Phospho-Akt1 (Ser473), Akt1, phospho-GSK3β (Ser9), GSK3β, β-catenin, CyclinD1, QCN5L2, PCAF, Acetyl-CBP, and CBP antibodies were purchased from Cell Signaling Technology. Oct4 and c-Myc antibodies were procured from Antibody Revolution. H3Ac, H3K9me3, H3K27me3, H3K36me3, H3K79me3, and H3 were obtained from Merck Millipore. KN93 (Cat. No. 422711), KN92 (Cat. No. 422709), Akt Inhibitor IV (Cat. No. 124011), β-catenin/Tcf Inhibitor V (Cat. No. 219334), and Wnt Pathway inhibitor XI (Cat. No. 681674) were purchased from Merck Millipore.

### Cell culture and oncosphere culture

A549, H1299, HCC827, H292, H1975, H358, and SK-MES-1 lung cancer cells were obtained from ATCC. SPC-a-1, LTEP-a-2, 95-C and 95-D lung cancer cells were purchased from cell bank of Chinese Academy of Science. SK-MES-1 cells were derived from squamous cell lung carcinoma, 95-C and 95-D cells were isolated from large cell lung carcinoma and others were harvested from lung adenocarcinoma. Primary lung cancer cells, ZRLC-1, ZRLC-3, and ZRLC-5 were isolated from tumor samples from lung adenocarcinoma patients. Primary normal lung cells, ZRNL-4, ZRNL-18 or ZRNL-19 were cultured from normal lung tissue, which had a more than 5 cm distance from the edge of lung cancer samples. All cancer cells were cultured with RPMI-1640 (GIBCO) supplemented with 10% fetal calf serum (GIBCO) in a 95% air, 5% CO_2_, 37°C humidified incubator. Oncospheres were cultured with DMEM/F12 (GIBCO) supplemented with 50 ng/ml EGF (Life Technology), 20 ng/ml bFGF (Life Technology), and 5% Bovine Serum Albumin (BSA, Life Technology), using ultra-low attachment cell plates (Corning).

### Ethics statement

Lung cancer samples were obtained from the Department of Pathology, with patient written informed consent. The Hospital's ethics committee approved all experiments. General information of 12 patients was listed as follows. Stage: IA 4, IB 2, IIA 2, IIB 4, III 1. Histological type: Squamous cell carcinoma 6, Adenocarcinoma 6. Smoking status: Smoking 8, Non-smoking 4.

### Flow cytometry assay and fluorescence-activated cell sorting

Cells were collected and washed with PBS, stained with CD133 antibody (PE, BD) or CD44 (APC, BD), washed again with PBS, and then examined by flow cytometry. Data were analyzed with FlowJ Software. Cells were FACS-sorted using a FACSAria (BD).

### Limiting dilution analysis

For the tumorigenicity of oncosphere cells, four-week-old NOD-SCID mice were used with the approval of animal ethics committee. Parental lung cancer cells or oncosphere cells were diluted serially to the desired cell doses (2.5×10^3^, 5.0×10^3^, 1.0×10^4^, 1.0×10^5^) and then separately subcutaneously injected in the left and right flanks of NOD-SCID mice. For the other xenograft tumor experiments, four-week-old nude mice (BALB/c nude) were used. Control or treated lung cancer cells (with CaMKIIγ overexpression, knockdown or inhibitor treatment) were diluted serially to the desired cell doses (from 1.0×10^3^ to 1.0×10^6^) and then separately subcutaneously injected in the left and right flank of nude mice. The number of tumors formed out of the number of sites injected was counted to determine the frequency of TICs, which was calculated using the ELDA software (http://bioinf.wehi.edu.au/software/elda/index.html).

### Stable knockdown & overexpression

For stable knockdown, lung cancer cells were transfected with control or CaMKIIγ shRNA using a lentiviral system, which was constructed in our previous study [14] and then cultured with puromycin (2.5 μg/ml) for two weeks. Next, transduced cells were seeded in 96-well plates at a single cell per well dose, for colony formation. The single colony wells were picked and digested for further culture and detection of knockdown. For stable overexpression, cells were transfected with control or wild-type CaMKIIγ using a lentiviral system, which was constructed in our previous study [14], and then cultured with blasticidin (10 μg/ml) for two weeks.

### Oncosphere formation assay

Counted cells were seeded in 24-well plates with conditional medium as described in the oncosphere culture section. The number of oncospheres was counted seven days later. Any oncosphere with more than 40 cells was counted.

### Real-time PCR, western blots, immunohistochemical staining, and co-immunoprecipitation

Real-time PCR, western blots, immunohistochemical staining, and co-immunoprecipitation (co-IP) were performed as described in our previous study [14]. Primers for real-time PCR were displayed in Table S1.

### Dual luciferase reporter assay

TOP, FOP, RBP-JK and GLI luciferase reporter plasmids were used to determine the effect of CaMKIIγ overexpression on Wnt, Notch, or Hedgehog pathways. These reporters (with firefly luciferase) were respectively co-transfected with pRL-null reporter (with *Renilla* luciferase) into control or CaMKIIγ overexpressed H1299 cells. Dual Luciferase^®^ Reporter 1000 Assay System from Promega corporation was used for the assay of firefly and *Renilla* luciferase. Firefly luciferase was normalized to *Renilla* luciferase activity.

### Chromatin immunoprecipitation

Chromatin immunoprecipitation (ChIP) was performed using SimpleChIP Enzymatic Chromatin IP kit (Magnetic Beads), according to the manufacturer's protocol (Cell Signaling Technology #9003). The ChIP-enriched DNA samples were quantified by real-time PCR.

### Oncomine^®^ data analysis

The Oncomine^®^ cancer microarray database (www.Oncomine.org">http://www.Oncomine.org) is an integrated bioinformatic platform that provides a well-organized collection of publicly available cancer microarray data [25]. CaMKIIγ (CaMK2G), OCT4, and MYC gene expression data, and clinical data from patients with lung cancers were obtained from the Oncomine^®^ database. All analyses were performed with R software (version 3.1.2). The correlation between CAMKIIγ and OCT4 or MYC was analyzed using Pearson correlation coefficient analysis. The correlation between CaMKIIγ expression and clinicopathological characteristics was analyzed using one-way ANOVA analysis or T test. Overall survival (OS) was calculated with the Kaplan–Meier method, and differences were compared by log-rank test. A *P* value <0.05 was considered statistically significant.

### Statistical analysis

Results are shown as mean ± *SEM*. Student's t test was performed to evaluate differences in experimental data. A *P* value <0.05 was considered statistically significant. Statistical analyses for Oncomine^®^ data were performed as described above.

## SUPPLEMENTARY MATERIALS FIGURES AND TABLE


