# Supplementary material for: Ca2+/calmodulin-dependent protein kinase IIγ enhances stem-like traits and tumorigenicity of lung cancer cells
Source: Oncotarget. 2015 Apr 19;6(18):16069–83. doi: 10.18632/oncotarget.3866 (PMC4599257; doi:10.18632/oncotarget.3866)
Supplement: Supplementary file 1 [file oncotarget-06-16069-s001.pdf]

Ca^{2+}/calmodulin-dependent protein kinase IIγ enhances stem-like traits and tumorigenicity of lung cancer cells

Supplementary Material

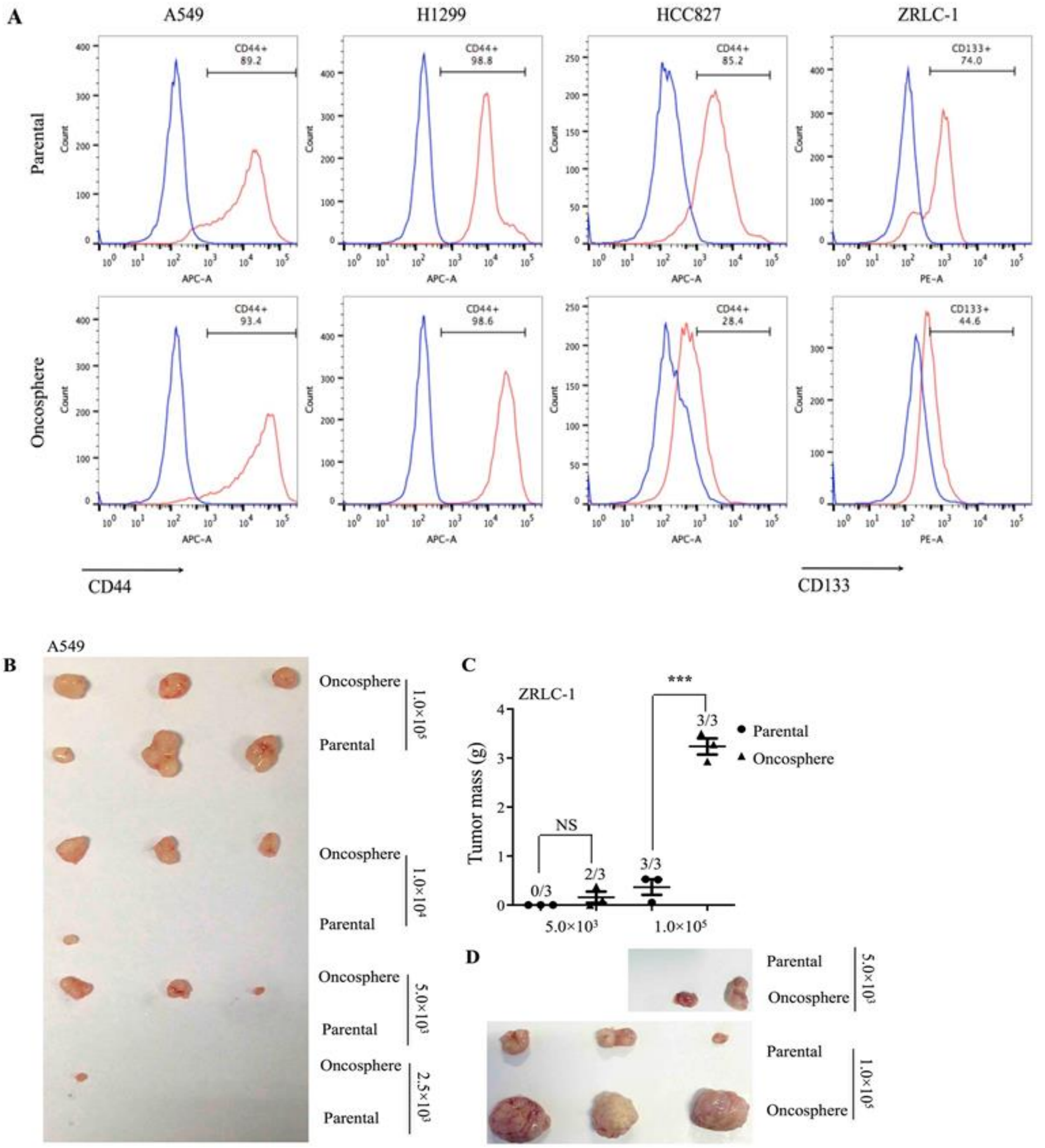

Fig.S1

(A) Representative histogram of CD44 or CD133 expression and quantification of CD44^{+} population in A549, H1299, and HCC827 parental or oncosphere cells, and CD133^{+} population in ZRLC-1 parental or oncosphere cells. The unstained control trace in blue is shown for gating.

(B) Representative image of tumors generated in mice that were injected with cells described in Fig.1D.
(C) ZRLC-1 parental or oncosphere cells were separately injected subcutaneously into NOD/SCID mice. Data are expressed as mean ± *SEM* of n=3 mice per group. ***p** < 0.05, NS no significance. Tumor incidence is displayed on the graph.
(D) Representative image of tumors generated in mice that were injected with cells described in Fig.S1C.

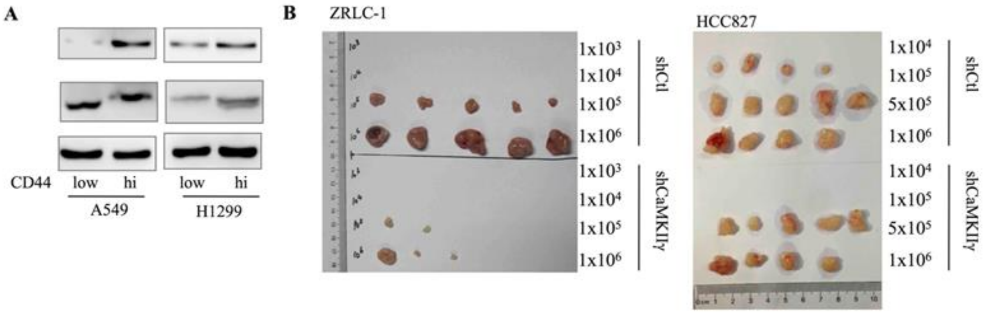

Fig.S2
(A) Detection of activated and total CaMKIIγ protein level by western blots in sorted lung cancer cells.
(B) Representative image of tumors generated in mice that were injected with cells described in Fig.2C.

A

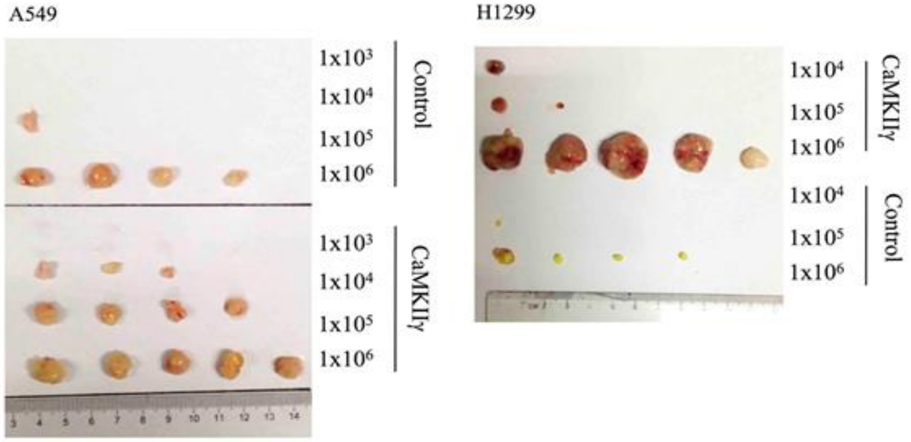

Fig. S3

(A) Representative image of tumors generated in mice that were injected with cells described in Fig.3C.

A

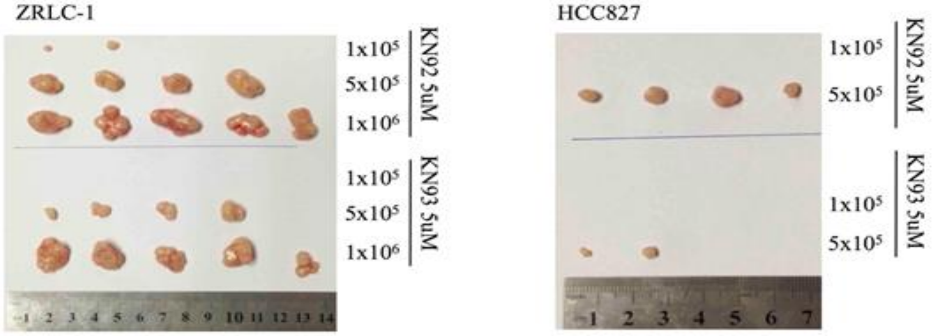

B

**TIC Frequency**	**ZRLC-1**	**HCC827**
**KN92 5uM**	1/164251 (95%CI: 1/402499-1/67028)	1/455120 (95%CI: 1/1212669-1/170809)
**KN93 5uM**	1/280490 (95%CI: 1/621072-1/126675)	1/1233156 (95%CI: 1/4874891-1/121976)

Fig. S4

(A) Representative image of tumors generated in mice that were injected with cells described in Fig.4D.

(B) TIC frequency of ZRLC-1 or HCC827 cells pre-treated with KN92 or KN93 is measured by LDA *in vivo*.

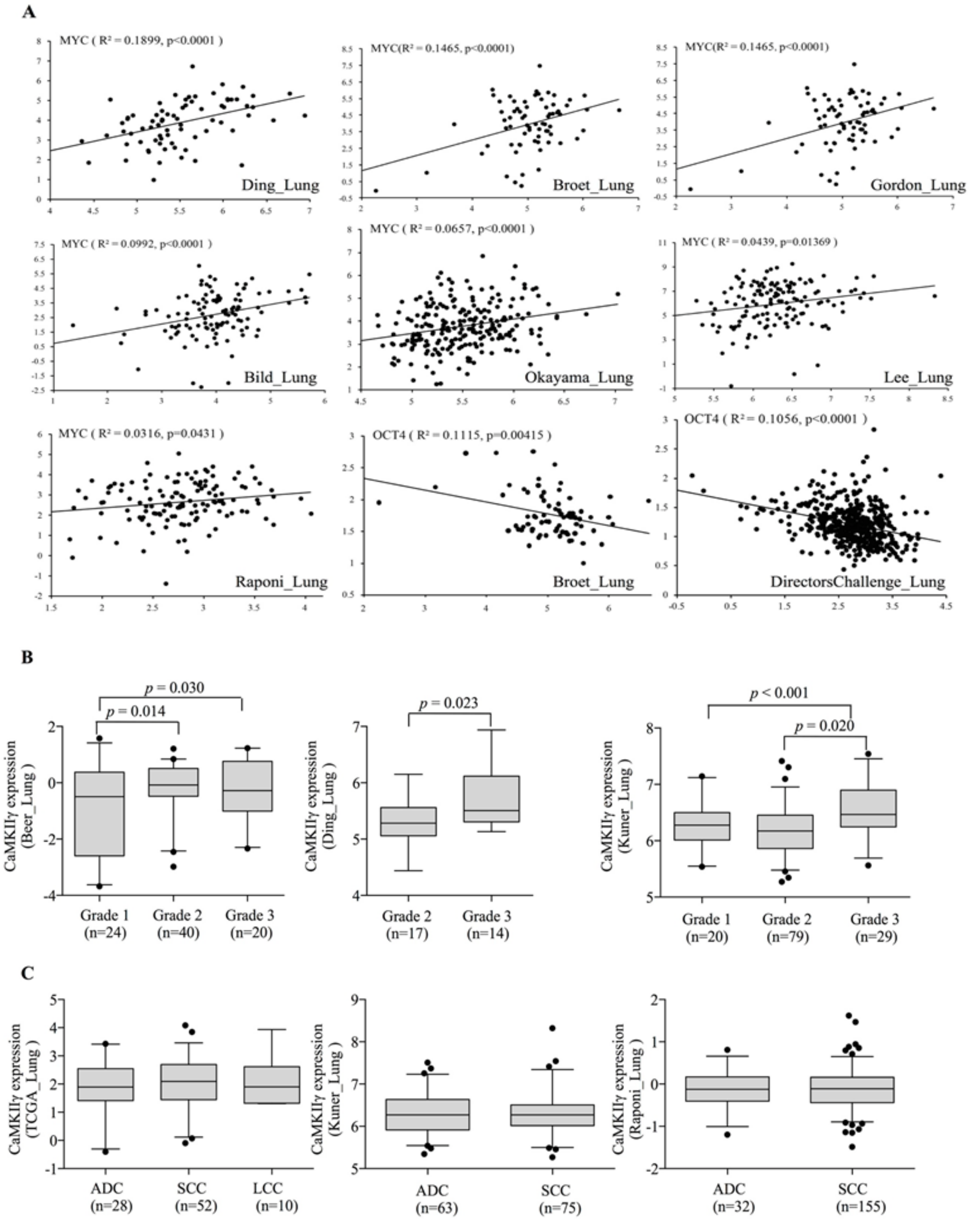

Fig. S5

(A) Correlation between CaMKIIγ and iPSC factors (OCT4, MYC) is analyzed from Oncomine® microarray data. X axis stands for CaMKIIγ expression and Y axis stands for OCT4 or MYC expression.

(B) Corrlation between CaMKIIγ expression and pathological grade is analyzed from Oncomine® microarray data.

(C) Corrlation between CaMKIIγ expression and cancer type including adenocarcinoma, squamous cell carcinoma and large cell carcinoma is analyzed from Oncomine® microarray data.

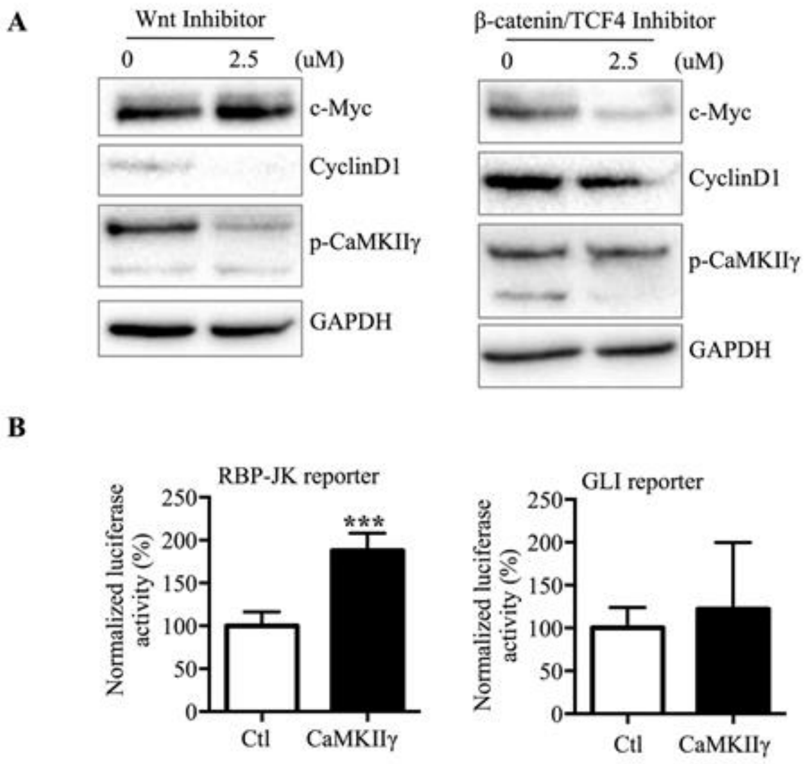

Fig. S6

(A) Detection of c-Myc, CyclinD1 and pCaMKIIγ in ZRLC-1 cells treated with Wnt or β-catenin inhibitor by western blots.

(B) Dual luciferase reporter assay of Notch pathway (RBP-JK) and Hedgehog pathway (GLI) in control or CaMKIIγ-overexpressed H1299 cells. Data are expressed as mean ± *SEM* of n=4 independent cell dishes per condition. *******$p < 0.001$.

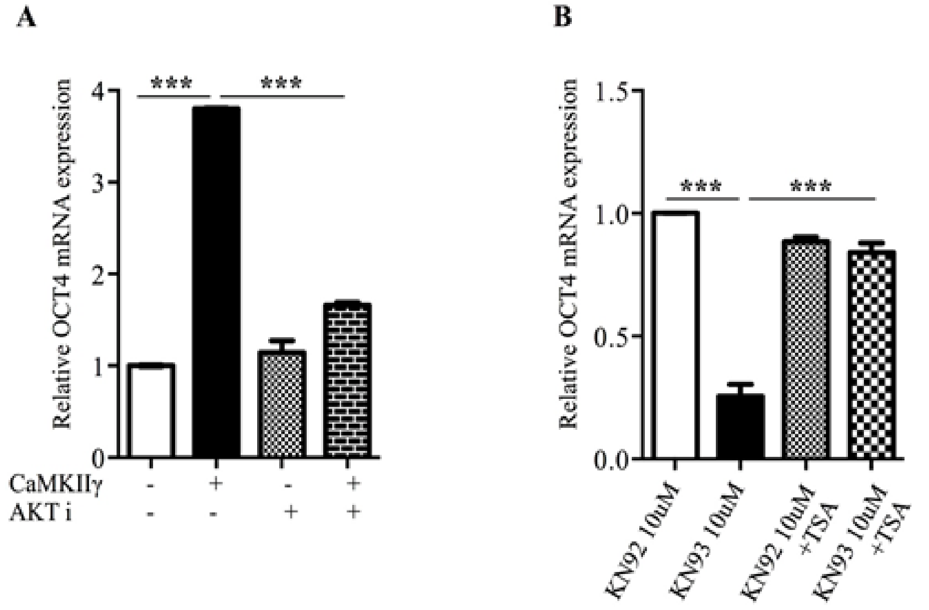

Fig. S7

(A) Relative gene expression of OCT4 in control or CaMKIIγ-overexpressed H1299 cells treated with Akt inhibitor. Data are expressed as mean ± *SEM* of *n*=3 independent cell dishes per condition. ****p* < 0.001.

(B) Relative gene expression of OCT4 in KN92- or KN93-treated ZRLC-1 cells cultured with TSA. Data are expressed as mean ± *SEM* of *n*=3 independent cell dishes per condition. ****p* < 0.001.

Table S1

Primers for realtime PCR and CH-IP PCR

Gene	Primer-F	Primer-R
OCT4	CTTGAATCCCGAATGGAAAGGG	GTGTATATCCCAGGGTGATCCTC
MYC	GGCTCCTGGCAAAAGGTCA	CTGCGTAGTTGTGCTGATGT
KLF4	CCCACATGAAGCGACTTCCC	CAGGTCCAGGAGATCGTTGAA
NANOG	TTTGTGGGCCTGAAGAAAACT	AGGGCTGTCCTGAATAAGCAG
CaMKIIγ	ACCCGTTTCACCGACGACTA	CTCCTGCGTGGAGGTTTTCTT
GAPDH	GGAGCGAGATCCCTCCAAAAT	GGCTGTTGTCATACTTCTCATGG
OCT4 (-2000)	GCATTCCGTTGGCTATTCTC	GATGTGCTTTGTTTAGTGGG
OCT4 (-1000)	TGTGCTTATGGCTGTTGATG	CCACTGTGCCCTGTTAGTTT
OCT4 (-100)	GCCACCACCATTAGGCAAAC	GCGAAGGGACTACTCAACCC
OCT4 (+1000)	AGAAAGCGAACCAGTATCGA	GCGCCGGTTACAGAACCACA
